# Classroom settings for visually impaired schoolchildren: A scoping review

**DOI:** 10.1371/journal.pone.0318871

**Published:** 2025-02-20

**Authors:** Nur Aresya Ahmad Najmee, Zainora Mohammed, Mohd Harimi Abd Rahman, Norliza Mohamad Fadzil, Arimi Fitri Mat Ludin, Rosilah Hassan

**Affiliations:** 1 Optometry and Vision Sciences Program, Centre for Rehabilitation and Special Needs Study, Faculty of Health Sciences, Universiti Kebangsaan Malaysia Kuala Lumpur, Kuala Lumpur, Malaysia; 2 Centre of Optometry Studies, Faculty of Health Sciences, Universiti Teknologi MARA, Shah Alam, Selangor, Malaysia; 3 Biomedical Science Program and Centre for Healthy Aging & Wellness, Faculty of Health Sciences, Universiti Kebangsaan Malaysia, Kuala Lumpur, Malaysia,; 4 Centre for Cyber Security, Faculty of Information Science and Technology, Universiti Kebangsaan MalaysiaBangi, Selangor, Malaysia; Universidad Santiago de Cali, COLOMBIA

## Abstract

Visually impaired schoolchildren require classrooms tailored to their visual abilities and needs, as outlined in existing literature detailing optimal modifications, recommendations, and guidelines. The study aims to review existing literature on classroom modifications for visually impaired schoolchildren, outlining recommendations for creating ideal classrooms within mainstream physical settings. After initial screening, 712 publications (698 from databases and registers, and 14 from other methods) were identified for detailed review, of which 17 were ultimately eligible for inclusion. Through a systematic review of PubMed, Scopus, and Web of Science databases, along with additional sources up to February 2024, this research analyzed articles published from 1999 to 2024. A qualitative, thematic analysis was conducted on the included articles. Criteria included peer-reviewed journals, theses, and conference papers focusing on classroom settings for visually impaired schoolchildren aged 7 to 18. Key questions addressed are: 1) What classroom settings suit the needs of visually impaired schoolchildren? 2) What recommendations are tailored to visually impaired schoolchildren to create a conducive classroom environment? Analysis of 7 journal articles and 10 other publications revealed two main themes: optimizing classroom configuration and enhancing visual comfort. Articles primarily focused on suggestions for classroom layout, particularly furnishing arrangement (41%, n = 7) and enhancing lighting conditions (41% n = 7). However, concerns were raised about standard furniture designs and the need for tailored seating arrangements to meet the visual demands of visually impaired schoolchildren. Conclusion: This review provides valuable insights into creating optimal classroom settings for visually impaired schoolchildren, ensuring equal learning opportunities in a supportive environment tailored to their needs.

## Introduction

Education is the foundation of individual and societal development, and its efficacy relies on equitable access to educational opportunities [1]. Inclusive education fosters an environment where all schoolchildren can participate without prejudice, emphasizing the need for schools to be sensitive to the diverse needs of schoolchildren with disabilities. Appropriate learning environments, personalized support from teachers, and access to visual aids are crucial for ensuring equal opportunities for visually impaired schoolchildren. As defined by the World Health Organization [2], visual impairment encompasses a visual acuity of 20/70 to 20/400 with optimal correction or a visual field of 20 degrees or narrower, indicating a spectrum of conditions leading to reduced vision and blindness.

### Inclusive classroom design

Schoolchildren with visual impairments face academic and social challenges that negatively impact academic performance, with their academic involvement remaining moderate [3,4]. The classroom environment emerges as a critical factor in achieving educational goals and fostering social inclusion, particularly for visually impaired schoolchildren. Previous studies underscore the significance of prioritizing the classroom environment alongside visual aids, therapy, and eyeglass prescriptions [5]. A prior study has concluded that incorporating the natural environment into buildings can positively influence the psychological, physical, and social well-being of the schoolchildren [6]. Seating position, furniture arrangement, and classroom layout are all important considerations when encouraging inclusive for schoolchildren with visual impairments. Optimal seating arrangements and adjustable layouts cater to a wide range of learning demands and promote independent navigation.

### Global classroom support

Nonetheless, gaps in support for visually impaired schoolchildren across school levels and curricula concerning physical classroom settings remain. In Korea, most special schools have appropriate lighting compared to general schools. However, both types of schools needed more comprehensive support for high-contrast mats and appropriate desks, indicating a need for improvement across the board [7]. In India, 72% of visually impaired schoolchildren face difficulties effectively viewing the chalkboard in classroom settings, necessitating strategies such as copying from peers or adjusting proximity to the chalkboard [8].

In contrast, early education settings across Australia demonstrate a remarkable effort to ensure that kindergarten classrooms meet satisfactory standards for visually impaired children [9]. Moreover, materials commonly utilized in early education settings seem to align with principles emphasizing features such as “bigger, brighter, contrast,” which are appropriate for visually impaired schoolchildren. However, as these children progress into older age groups, the adequacy of the physical classroom environment becomes an intriguing area for investigation, necessitating more evaluation.

In Malaysia, the implementation of various government policies and regulations to support children with disabilities in the educational system, including The Persons with Disabilities (PWD) Act of 2008, the 2013 Education (Special Education) Regulations, and the National Education Blueprint (2013–2025) are among the key initiatives emphasizing quality improvement and inclusive education [10]. Nonetheless, specific attention to classroom settings for visually impaired schoolchildren is lacking within these frameworks, and research in special education remains relatively limited within this region.

Furthermore, the existing regulations governing standard classroom settings in Malaysia have addressed the needs of normal-sighted, potentially overlooking the unique needs of visually impaired schoolchildren. For example, the Economic Planning Unit (EPU) provides detailed specifications for classroom space, including size and occupancy, with the recommended floor areas per student in primary and secondary schools ranging from 7.8 to 18.8 square meters and 10.5–21.0 square meters, respectively [11]. Consequently, secondary schools should provide at least 2.25m² of classroom space per student. However, the direct applicability of these criteria to visually impaired schoolchildren remains uncertain.

Regarding lighting, the minimum level of classroom lighting in Malaysia’s low-vision schools falls short of the recommendations outlined in the Malaysian Standard MS 1184:2014 for individuals with special needs, including visual impairment [12]. Recommendations have been proposed to improve lighting conditions by removing obstacles to natural light or by adding artificial lighting to improve the performance and comfort of schoolchildren during teaching and learning activities [13]. However, the extent to which lighting standards and strategies meet the needs of visually impaired schoolchildren requires investigation as the standard classroom lighting for normal student writing and reading on the blackboard is 500lux and 300lux for student-teachers talking and paying attention [6].

Hence, it is crucial to establish an optimal classroom environment for visually impaired schoolchildren that accommodates their residual vision and visual function characteristics. Despite numerous global guidelines derived from government policies and school practices, there has yet to be a comprehensive effort to synthesize these resources.

### Aims

The primary purpose of this review is to systematically arrange and synthesize existing classroom guidelines and recommendations, serving as invaluable references for policymakers and researchers striving to design inclusive classroom settings tailored to the unique needs of visually impaired schoolchildren.

## Methods

In conducting this scoping review, the methodological framework developed by Arksey & O’Malley [14] was adopted. The reporting of the search process was refined following the checklist outlined in the Preferred Reporting Items for Systematic Reviews and Meta-Analyses extension for Scoping Reviews (PRISMA-ScR) Checklist refer to Supporting Information (S1 File). The protocol of this review was pre-registered on the Open Science Framework (OSF) platform: http://osf.io/z2sdt and subsequently published detailing the planned methodology, search strategy, and analysis approach [15].

This study systematically conducted a five-stage process, including formulating the research question, identifying relevant studies, selecting studies, organizing and analyzing data, synthesizing, summarizing, and presenting findings. The choice of a scoping review methodology aimed to identify gaps in the literature regarding interventions, extending beyond those already proven effective or meeting predefined quality assessment criteria [16].

### Identifying research questions

This review addressed the primary question- Are there standardized physical classroom settings and recommendations regarding measurement, layout, lighting, contrast, and optimal positioning tailored specifically for visually impaired schoolchildren?

The primary question leads to two detailed sub-questions to be answered in this review:

1. How can the arrangement of furniture, seating positions, and classroom space optimize the classroom configuration to help in the mobility and navigation of visually impaired schoolchildren?
2. How do variations in lighting, color, and contrast affect visual perception and comfort, particularly regarding material legibility for visually impaired schoolchildren?

### Search terms and search strategies

A literature search was executed in December 2022, and an initial scoping search was undertaken to assess the viability of the subsequent review. The search strategy was formulated and independently validated by three reviewers. Studies were limited to those published in English. A systematic search was conducted on selected databases of research articles published from January 2000 to December 2020. A subsequent search was then executed in February 2024 to encompass more recent literature published up to December 2023. This final phase included article search for supplementary pertinent literature, alongside a search of gray literature conducted via Google. The complete search strategy, including all search terms and database-specific syntax, is provided in Supporting Information (S2 File).

### Study selection

The study selection criteria for this academic paper were established through a multi-stage review process. The targeted population comprised studies concentrating on visually impaired schoolchildren 7–18 years old [17,18]. The schoolchildren's range encompasses both mainstream and specialized educational settings. The education begins with six years of Primary Education (ages 7–13), followed by three years of Lower Secondary Education (ages 14–16), and concluding with two years of Upper Secondary Education (ages 16–18).

The inclusion criteria for the setting involved studies conducted in traditional classroom environments, with a scope extending to diverse geographical locations and educational systems. Eligible studies were those that investigated or discussed interventions related to lighting, contrast, physical measurement, layout, and seating position in classrooms for visually impaired schoolchildren to optimize the classroom environment for this demographic. Moreover, selected studies that assessed the impact of classroom settings on the educational experience, participation, and academic outcomes of visually impaired schoolchildren, and also included research reporting on their preferences and feedback, particularly regarding classroom design. In terms of study design, the criteria covered peer-reviewed empirical studies, including experimental, quasi-experimental, observational, and qualitative research, as well as systematic reviews and meta-analyses focused on the specific topics pertinent to the study. The publication status criteria included published articles in peer-reviewed journals, as well as theses, dissertations, and conference proceedings demonstrating relevance and rigor in research. The temporal scope for eligible studies spanned from 1990 to 2024. Studies primarily investigating interventions or exposures related to assistive technologies designed for visually impaired schoolchildren will not be considered. Furthermore, studies conducted among schoolchildren with multiple disabilities and mental illness have been excluded.

### Selection of included publication

The first and second authors identified all of the studies using database searches. The results were then evaluated for eligibility by reviewing each listed study’s title and abstract. The authors retrieved the full text of possibly relevant studies for further screening of their suitability based on the review question and inclusion criterion. Full-text versions of articles meeting the final inclusion criteria were obtained. The details of these selected articles were then recorded and managed using Rayyan.ai, a systematic review software tool. Each study was evaluated by labeling it as include, exclude, or maybe. Any that were rated as maybe were discussed among the authors. If there was a disagreement, a third reviewer was consulted to reach a final determination regarding the inclusion of the article.

### Charting and collating the data

The process of data extraction involved collecting various details from the included articles, such as publication year, study objectives, target demographics, and intervention of the study. Subsequently, a qualitative thematic analysis was undertaken to explore the impact of classroom environmental factors such as layout, lighting, contrast, and seating arrangements on creating optimal learning conditions for visually impaired schoolchildren. Initially, codes were generated and then systematically reviewed and categorized into potential themes, which were subsequently refined through iterative analysis [19]. All authors contributed to determining the most suitable approach for interpreting the data.

## Results and discussions

Initially, through a comprehensive search of databases and registers, a total of 1,868 articles were identified: PubMed (n = 444), Scopus (n = 996), Web of Science (n = 406), and Electronic Educational Research (n = 22). Additional records were extracted through other methods including websites (n = 3), organizations (n = 7), books (n = 4), and ProQuest Dissertations & Theses Global (n = 3).

The preliminary filtering process excluded 1,070 articles from databases and registers based on predefined criteria including non-English publications, conference abstracts lacking full text, and studies unrelated to classroom settings or visual impairment in schoolchildren. Following this, 100 duplicate records were removed, yielding 698 articles for screening. Of these 698 articles, 465 were excluded during the title and abstract screening phase, and 58 reports were not retrieved. Similarly, from the 17 reports identified through other methods, 3 reports were not retrieved (1 from the website, 2 from books/thesis). This left 175 full-text articles from databases and 10 reports from other methods to be assessed for eligibility. After assessment, 168 articles from databases were excluded due to wrong setting (n = 28), wrong population (n = 48), wrong intervention (n = 46), and wrong outcome (n = 46). From the other methods, 4 reports were excluded due to content redundancy (n = 2) and reports being too short (n = 2). The final analysis included 17 studies (7 research articles from databases and 4 from guidelines, 3 from chapters in books, 2 from theses, and 1 website).

**Fig 1:**
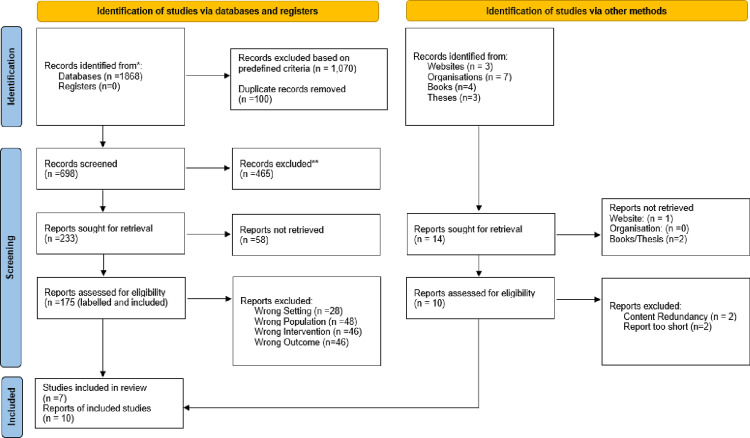
PRISMA flow diagram for scoping review process. Systematic screening identified 1,868 records from databases. After removing duplicates and screening, 17 studies were included in the final analysis.

A comprehensive PRISMA flow diagram detailing the screening and selection process, with additional details provided in Supporting Information (See Fig 1).The complete list of all identified studies with their resources, including reasons for exclusion, is available in Supporting Information (S3 File).

### Descriptive summary of the studies

This review encompasses a total of 17 articles, presenting findings from 7 scholarly articles, and 10 other publications. The overview of these articles is presented in Table 1. Spanning the years 2001–2023, the included research studies were conducted in Canada (n = 2), India (n =  2), followed by the United States of America (n =  1), Australia (n =  1), and Nigeria (n =  1). The study designs varied, featuring four cross-sectional and three systematic reviews. Most of these studies were exclusively focused on the low vision and visual impairment group, with participants ranging from 6 to 18 years old.

**Table 1:** Descriptive Overview of Selected Studies.

Author	Year	Country	Study design	Population	Age	Intervention
**Research Studies**						
Negiloni	2018	India	Cross-Sectional	Low vision School Children	5–17	Evaluate the classroom environment for visually impaired school children Provide recommendations to minimize visual strain, especially in regular school settings.
Negiloni	2017	India	Cross-Sectional	Classroom Measurement	10–18	Assessing the distance and near visual acuity demand in Indian school classrooms and comparing them with the recommended vision standards.
Amiebenomo	2023	Nigeria	Cross-Sectional	Visually Impaired School Children	6–18	Analyse the burden of blindness and vision impairment in Nigerian schoolchildren. Determine the proportion of students meeting the visual acuity (VA) demand for their classroom.
Lovie Kitchin	2001	Australia	Cross-Sectional	Low Vision Students	7–18	Exploring the relationship between clinical vision measures and reading performance in children with low vision.
Cox PR	2001	Canada	Systematic Review	Visual Impaired Students	6–18	Implementing strategies to integrate students with visual impairments into general education settings.
Saskatchewan Learning	2003	Canada	Systematic Review	Visually Impaired Students	6–18	Offering insights into how a student utilizes their remaining vision in various learning environments. Provide recommendations for implementing classroom adaptations.
Daniela Gissara	2023	US	Systematic Review	Visually Impaired School Children	6–18	Describe the general recommendation for activities and materials for students with low vision (change environment and learning material)
**Other publications**						
DOSH	2018	Malaysia	Guidelines	All Students		Highlight the importance of inclusive lighting design that takes into consideration the needs of visually impaired individuals Address the need to minimize glare to ensure a comfortable and safe working environment for everyone
EPU	2015	Malaysia	Guidelines	All Students		Provide standard classroom size and student capacity for all classrooms in Malaysia
NIBS	2014	US	Guidelines	Visually Impaired Students		Prioritize creating an inclusive and accessible learning environment for visually impaired people such as uniform illumination, contrast, and color coding.
MS 1184	2015	Malaysia	Guidelines	Low Vision Students		Support individuals with visual impairments, creating a conducive environment involves enhancing orientation and mobility.
David Mitchell	2007	UK	Book Chapter	Special Students		Provide a quality indoor physical environment that enables conducive learning in inclusive education
Wong Huey Siew	2006	Malaysia	Book Chapter	Visually Impaired Students		Analysing the learning environment for visually impaired students in Malaysia
Arter	1999	UK	Book Chapter	Visually Impaired Students		Provide information on major adaptations of the school environment to meet the needs of visually impaired students
P.Alagappen	2020	Malaysia	Thesis	Visually Impaired Students		Concentrates on the design strategies essential in a school setting, with a particular emphasis on classroom design tailored specifically for visually impaired students.
Norsafiah Norazman	2019	Malaysia	Thesis	All Students		Create a tool designed to evaluate the performance rating of school buildings, with a specific emphasis on assessing the current conditions of classrooms within secondary school buildings.
Carmen Willings	2019	UK	Website	Visually Impaired Students		Provide modification for classroom arrangements to accommodate blind and visually impaired students.

*Data extraction was conducted by multiple reviewers using Rayyan.ai, as detailed in Methods section 2.4. The process involved independent review and collaborative resolution of any discrepancies.

Other publications encompassed standard guidelines from government departments, and institutions, websites, and book chapters. The standard guidelines obtained were primarily from Malaysia (n = 3) and the United States (n = 1), and published between 2014 and 2018. These guidelines covered occupational safety for lighting, development and planning in buildings for special needs, and the design of visual environments, buildings, and classrooms for individuals with low vision. Moreover, this review also incorporates insights from book chapters (n = 3) and theses (n = 2) related to designing optimal classrooms. The first book emphasizes the quality of the indoor physical environment for inclusive education [20], while the second discusses the learning environment for visually impaired schoolchildren in Malaysia [21]. The third book focuses on classroom adaptations to meet the needs of visually impaired schoolchildren in mainstream settings [22].

Additionally, a thesis focuses on design strategies, particularly in classroom design for visually impaired schoolchildren, employing multisensory architecture for a safer and more comfortable learning environment [11]. Nonetheless, another thesis evaluated the performance rating of secondary school buildings, with a particular focus on assessing current classroom conditions considering factors such as accessibility features, lighting, contrast, acoustic environment, and availability of tactile and auditory resources to create optimal settings for visually impaired schoolchildren [22]. Meanwhile, a website addressed the strategies for teaching and learning of visually impaired schoolchildren [23].

### Prevalence of classroom design factors for visually impaired schoolchildren

Across all selected articles, classroom layout in terms of furniture arrangement (47%) and seating position (41%) of schoolchildren are the criteria that are frequently cited. Meanwhile, lighting conditions (47%) and glare (23%) are also prominently mentioned to enhance the visual comfort of visually impaired schoolchildren in classroom settings. The recommendations for classroom settings corresponding to each criterion from the selected studies are compiled and presented in Table 2.

**Table 2:** Recommendation for Classroom Settings from Selected Studies.

No.	First author, Year	Aim	Classroom Settings				
			Layout	Contrast	Size	Lighting	Seating Position
[5]	Negiloni, (2017)	Evaluate the distance and near Visual Acuity (VA) demand in Indian school classrooms and compare the recommended vision standards	X	X	X	X	
[8]	Negiloni, (2018)	Evaluate the classroom environment of low vision in mainstream settings, and provide recommendations to reduce visual stress	X	X	X		
[11]	Norsafiah Norazman, (2019),	Develop a tool to assess the performance of secondary school buildings, focusing on evaluating classroom conditions.	X	X		X	
[20]	David Mitchell, (2007)	Provide a quality indoor physical environment that enables conducive learning in inclusive education		X	X		X
[21]	Wong Huey Siew Institute Social Malaysia, (2006)	Evaluate the perspectives of visually impaired students and their teachers on the learning environment in Setapak, Malaysia.		X	X	X	
[22]	Christine Arter (1999)	Offer teachers guidance on adapting the school environment for visually impaired students			X	X	X
[23]	Prabu Alagappen, (2019)	Propose designing strategies tailored for visually impaired students in school environments.			X		
[24]	Carmen Willings, (2019)	Inform classroom modifications to accommodate blind and visually impaired students in mainstream settings			X		X
[29]	Saskatchewan Learning, (2003)	Provide information to the teachers regarding the needs of students who require intensive support.			X		X
[31]	Daniela Gissara, (2023)	Provides information about low vision; the causes and implications in learning and development			X		X
[33]	MS 1184 Department of Standards Malaysia, (2014)	Enhance orientation and mobility for visually impaired people through visual contrast in building interiors and implementing tactile ground surface indicators.	X		X		X
[34]	Standards and Costs Committee, Economic Planning Unit, Prime Minister’s Department Malaysia, (2015)	Provide standard classroom size and student capacity for all classrooms in Malaysia	X	X		X	X
[37]	National Institute of Building Sciences, (2015)	Provide recommendations for architects and designers to optimize environmental features, contrast, and daylighting control, ensuring safe navigation and visual comfort for individuals with low vision.	X		X		X
[41]	Amiebenomo, (2023)	Estimate the burden of vision impairment in Nigerian schoolchildren and determine the proportion of students meeting the visual acuity (VA) demand in their classroom	X	X	X	X	
[42]	Department of Occupational Safety and Health, Ministry of Human Resource Malaysia, (2018)	Provide lighting requirements for standard classrooms in Malaysia	X	X	X		X
[48]	Cox PR, (2001)	Strategies to include visual impairments students in general education settings.	X		X	X	X
[49]	Lovie Kitchin, (2001)	Examine the relationships between clinical vision measures and reading performance in children with low vision	X		X	X	X

The qualitative thematic analysis revealed insights into optimal classroom settings that were categorized under two primary themes: Classroom Arrangement and Visual Comfort. The resulting key themes are outlined in Table 3. The specific classroom criteria within these themes are customized to meet the needs of visually impaired schoolchildren, ensuring adaptability for implementation in both mainstream and special education settings.

**Table 3:** Overview of Key Themes.

Classroom Settings	Criteria	Applied for	Studies
Classroom Arrangement (optimizing classroom configuration)	Functional Placement and Organization 1.1 Furniture Arrangement 1.2 Capacity and Size 1.3 Seating Position	VIS, LVS AS, VIS, LVS AS, LV, BLIND, VIS	[20–24,29,31] [11,21,23,34] [2,5,11,21,23,41]
Visual Comfort (minimizing glare and maximizing contrast)	Lighting 1.1 Lighting Condition (Specific Task Requirement/Adjustable) 1.2 Glare and Shadow Management Color and Contrast 2.1 Basic Principal Color and Contrast 2.2 Color and Contrast Materials 2.3 Color and Contrast for Interior Space	AS, LVS, VIS, BLIND, SS AS, LVS, VIS, BLIND, SS VIS, BLIND LVS, BLIND, VIS, SS LVS, BLIND, VIS	[8,20,24,31,33,37,42] [20,23,29,31,33,37] [23,24,31,33,48] [22,49] [22,24,33,37]

*AS = All Students, VIS = Visually Impaired Students, LV = Low Vision, SS = Special Students.

### Classroom arrangement

This theme focuses on optimizing classroom configuration to enhance functionality and ensure safety, accessibility, and independence. It involves strategic placement and organization of the furniture, considering the capacity and size of the room, and determining optimal seating positions tailored to the needs of schoolchildren with visual impairments, taking into account their visual abilities

### How can furniture, seating arrangement, and classroom layout optimize the mobility and navigation of visually impaired schoolchildren?

#### Furniture selection.

In creating optimal classrooms for visually impaired schoolchildren, several key factors are considered including thoughtful furniture selection, clear organization, appropriate classroom sizing and capacity, as well as strategic seating arrangements and layout. In educational settings, classroom furniture commonly comprises desks, tables, seating arrangements, and bookshelves, often adhering to standards set by relevant regulatory bodies or educational authorities [25].

Furniture is essential in creating conducive teaching and learning environments, influencing behavior, and fostering creativity [26].(Chlopek & Niedbala, 2023). Studies have suggested recommendations for optimizing classroom furniture to meet the diverse physical needs of visually impaired schoolchildren. This includes using flexible desks, along with adjustable height tables and amenities like reading stands to enhance flexibility and physical comfort, as well as promote proper posture [20,22–24,26]

These recommendations emphasize the importance of adjustable tables or reading and writing stands with spacious work surfaces to prevent visual fatigue caused by prolonged stooping over work. Desks that accommodate visually impaired and blind users are suggested to have a length of 1.4 m [23]. Adequate storage solutions are also necessary for organizing papers, bulky Braille books, and other necessary equipment [22,24]. Additionally, chairs in the classroom should offer maximum placement and flexibility while considering ergonomic factors, to ensure that schoolchildren maintain flat feet on the floor. Tables should be positioned at heights that provide upper body support and facilitate movement, avoiding excessively low or high placements [23].

Previously, there is a detailed and specific design for kitchen furniture aimed at enhancing safety for visually impaired individuals has been developed [27]. However, to date, classroom furniture primarily revolves around adapting and recommending standard designs suitable for all schoolchildren, rather than being specifically tailored to the visually impaired. Additionally, a survey conducted at a secondary special school for visually impaired schoolchildren in Malaysia highlighted outdated desks and chairs, underscoring the importance of annual inspections [21,28].

#### Classroom arrangement.

Creating an optimal learning environment and organizing supplies requires meticulous planning to ensure ease of navigation and identification while minimizing distractions and seamless integration of visual learning cues. Moreover, avoiding overcrowding the classroom and blocking walkways while identifying and removing potential hazards further fosters a safer and more accessible learning environment for visually impaired schoolchildren [20]. This can be facilitated by integrating sensory glass sliding entrance doors of the classroom, with highlighted door edges [23,29].

The recommendations are aligned with a previous study, on interior design for visually impaired individuals. This study emphasizes that design must prioritize the appropriate arrangement and safe distance of furniture and equipment to prevent hazardous obstacles, enhance the usability of various spaces, and promote ease of navigation to enhance confidence in independent mobility [30].

Moreover, additional studies underscore the importance of minimizing clutter, positioning the teacher’s desk appropriately, and thoughtfully placing trash bins, all recognized as recommended practices [21,22]. Teachers should ensure that these schoolchildren are familiar with furniture placement and should promptly notify them of any changes to promote comfort and autonomy [20,29,31]

Additionally, implementing clear separation between activity and learning areas, along with labeling systems, will aid in item location and literacy promotion [24]. Explanation regarding the layout and storage arrangements at the beginning of each school year is also useful [22,24]. Studies reported that familiarity with the layout and storage arrangements helps visually impaired schoolchildren navigate independently, promoting independence and enhancing learning outcomes.

#### Classroom layout.

The classroom layout is primarily shaped by the seating arrangements and room dimensions, with notable differences between designs for visually impaired schoolchildren and their sighted peers. Traditional classroom layouts, which prioritize space optimization and effective communication between schoolchildren and teachers, often incorporate seating arrangements like grids, clusters, and U-shaped arrangements [32]. These layouts are recognized by educational standards for fostering adaptable and conducive learning environments [11]. There are commonly two types of seating arrangements recommended for visually impaired schoolchildren; one for learning and the other for discussion [22,23]. Learning settings typically utilize a traditional grid spatial arrangement while discussion requires the children to face each other to form a symmetrical arrangement.

Concerning the classroom capacity and size of the classroom, the Economic Planning Unit (EPU) specifies that in Malaysia, each classroom can accommodate up to 35 students with a minimum area of classroom per student at 2.25m² [11,33,34]. However, for visually impaired students, the capacity in standard-size classrooms typically ranges from 3 to 6 students aiming to facilitate tailored support and individualized attention [21,23].

**Fig 2:**
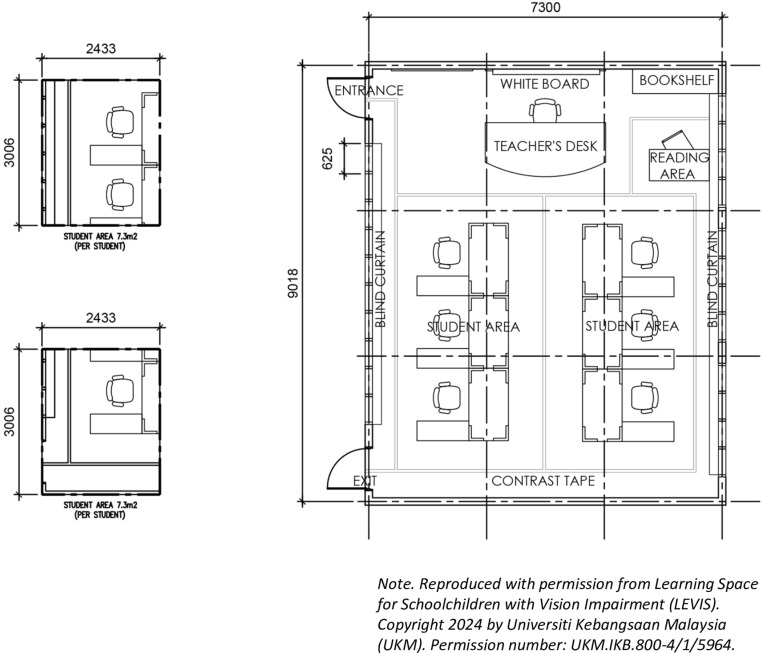
Optimal classroom layout. Diagram showing recommended furniture arrangement and seating positions within 45.96 ±  6.98 m² space for visually impaired students (LEVIS): UKM.IKB.800-4/1/5964.

Empirical measurements from a special blind school in Setapak, Malaysia, resulted in a minimum area of 45.96 ±  6.98 square meters, corresponding to 7.2 square meters per student [35]. The finding aligns with classroom size standards for visually impaired schoolchildren in Indonesia [36]. All the recommendations derived from published data were synthesized and adapted into actual classroom sizes in one of the special schools in Malaysia. The recommended classroom layout for visually impaired school children (See Fig 2) is reproduced with permission from Learning Space for Schoolchildren with Vision Impairment (LEVIS) UKM.IKB.800-4/1/5964.Arranging furniture in small groups among visually impaired individuals has been suggested to encourage seamless communication [21,23]. Other studies indicate that classroom layouts for visually impaired schoolchildren should focus on facilitating easy identification, promoting item location, enhancing navigation, and fostering literacy through strategic furniture placement [22,24,37,38].Additionally, seating these children near outlets is also crucial if they use electronic magnifiers or similar devices, with additional attention given to accommodating their equipment [23].

A previous study suggests that seating arrangements can be made randomly in discussion settings to promote active learning and increase predictability among the visually impaired or strategically based on visual impairment criteria [39]. Specific recommendations were identified based on the severity of visual impairment, to alleviate visual stress when viewing the chalkboard. For schoolchildren with mild impairment, a maximum seating distance of 4.3 meters from the chalkboard is recommended, with a visual task size of 3 cm for lowercase letter writing. For those with a visual acuity range of 3/60–6/60, a maximum viewing distance of 85 cm to 1.7 meters is advised, with a visual task size of 4 cm [8]. However, determining the seating distances based on the level of impairment should consider their visual demands in the classroom, involving simple adjustments in classroom parameters, like the size of the teacher’s writing on the chalkboard and viewing distance [5].

Additionally, evaluating visual task demand based on chalkboard writing requires consideration of visual acuity reserve. Thus, understanding visual demand and reserve is crucial in determining seating for visually impaired schoolchildren. Several factors need to be considered, such as classroom size, magnification of viewed materials, contrast, and illuminance of the classroom environment and learning materials which can influence visual demand and reserve [40].

A significant concern arises regarding classroom seating arrangements, where uniformity among visually impaired schoolchildren still needs improvement. Surveys conducted in a blind school in Malaysia revealed a lack of uniformity in group student arrangements, underscoring the importance of determining appropriate positions for visually impaired schoolchildren. Discrepancies from recommended seating standards were also noted in classroom settings in India and Nigeria, mainly longer distances observed in secondary school classrooms in Nigeria [5,8,21,41].

### Visual comfort

This theme emphasizes recommendations for optimizing visual comfort through considerations of lighting, colour, and contrast. The lighting strategies aim to achieve optimal illumination levels, mitigate glare, and address various visual impairments to enhance visual comfort. Similarly, the discussion on colour and contrast underscores the importance of maximizing contrast, selecting appropriate colours, and minimizing glare to improve readability and visibility, thereby promoting visual comfort, particularly for visually impaired schoolchildren.

### How do variations in lighting, colour, and contrast affect visual perception and comfort, particularly regarding material legibility for visually impaired schoolchildren?

#### Lighting.

The lighting condition encompasses a spectrum of considerations, ranging from general illumination to task-specific lighting. Recommended lux levels vary depending on the nature of tasks, and range from 200 to 1000, ensuring adequate illumination, glare limitation, uniformity, and suitable colour rendering [33,42]. Effective lighting design significantly influences environmental perception, making it crucial for creating conducive work environments, especially for visually impaired individuals [32]. Lighting designs for visually impaired schoolchildren should be tailored to accommodate various tasks, and areas, and address different levels of visual impairment [20]. Ensuring appropriate lighting levels, magnification, and material contrast is paramount for improving visual comfort, particularly for schoolchildren with reduced visual acuity [8,31].

In educational settings, daylight plays a significant role in positively influencing learner performance. It is essential to optimize the utilization of natural daylight while complementing it with artificial lighting as necessary [20,24]. Classrooms typically require 300 lux for regular sessions and 500 lux for evening classes to promote optimal visibility and learning [42]. This range aligns with recommendations for different types of classrooms as stated in the Code for Lighting London suggesting levels between 300-500 lux [43].Nonetheless, the minimum light level required for the visually impaired person varies based on the task, with horizontal surfaces indoors requiring at least 100 lux and tasks with small details or low contrast requiring 1000 lux for enhanced clarity [33]. To comply with energy regulations, the Lighting Power Density (LPD) thresholds need to be increased in areas frequently utilized by visually impaired individuals, reflecting the necessity for higher illumination levels for this population [37]. The minimum lighting requirements based on areas are tabulated in Table 4.

**Table 4:** Minimum Lighting Requirement for Different Areas.

Area	E minimum (lux)
Chalkboard and student desks	150–300 lux
Habitable spaces	300–500 lux
Offices: writing and typing	500 lux
Conference and meeting room	500 lux
Classrooms: tutorial rooms	300 lux
Classroom for evening classes	500 lux
Visual tasks with small details or low contrast	1000 lux

The classroom brightness standards may change with breakout spaces and display wall adjustments, even though numerous building codes advocate for a consistent brightness level of 55-foot candles [20]. In India, the Bureau of Indian Standards has advised to maintain the optimal illuminance levels on the chalkboard and student desks within the range of 150–300 lux. However, instances have been documented where these standards were not met [40]. In Malaysia, a previous study reported that the morning lighting level in a special school for visually impaired schoolchildren was measured as substandard [12].

Thus, proper interior lighting and uniform distribution should be the foundation when designing lighting installations for visually impaired individuals to enhance security, easier differentiation of architectural elements, and aids for orientation and mobility [32]. This can be achieved by integrating various lighting types such as incandescent lighting with fluorescent systems [23,24,31,33,] It is advisable to select ambient lighting with varying brightness levels and colors, preferably with a color temperature that suits the environment to aid the learning physiologically [44,45].

Artificial light such as fluorescent or compact fluorescent bulbs are energy-efficient lighting options that can be effectively utilized in conjunction with dimmer switches to regulate light levels. This flexibility in controlling illumination is particularly beneficial for mitigating photosensitivity in individuals with visual impairments [46]. Additionally, light-emitting diodes (LEDs) can also be employed in specific areas or for particular tasks, such as reading, sewing, and crafting, as they provide focused illumination ideal for close-up activities [47]. A well-designed lighting setup involves properly locating light sources and combining both natural and controllable artificial light, ensuring adequate illumination in workspace while minimizing glare or excessive contrast [36,44].

#### Glare and shadow management.

Glare presents significant challenges for visually impaired schoolchildren, hindering task performance and causing visual discomfort. It occurs due to excessive luminance contrast between interior elements, especially windows, light fixtures, and reflective surfaces. Specular reflections from interiors such as chalkboards and student’s desks can lead to glare spots, causing discomfort and disability glare. Even though sunlight or natural light is considered the ideal source, it can also cause glare and shadows both indoors and outdoors especially when coupled with excessive fluorescent lighting that varies across different areas [45].

Strategies to mitigate glare in the classroom include strategically placing windows and light fixtures, using curtains to balance lighting, and shielding or shading light sources [23,31,33,37]. Shielding techniques such as adjustable blinds or curtains with automatically dimming luminaries, can help control both artificial and natural lighting. However, venetian blinds should be avoided, as they can induce pattern glare.

Additionally, minimizing luminance contrast between interior elements and adopting a combination of direct and indirect lighting can help reduce glare [20,37]. Walls and ceilings should be painted in plain light tones with matte finishes to diffuse light and prevent glare [33].Incorporating multiple daylight sources and higher ceiling heights can achieve a balanced illumination level. However, reliance solely on daylight may lead to insufficient lighting for visually impaired schoolchildren, compromising the learning environment. The effective depth of daylight penetration typically reaches around 15 meters (approximately 45 feet) in the absence of skylights or light shelves.

Strategic seating arrangements and building design considerations, such as an Aspect Ratio greater than 3.0 to enhance daylight penetration and preferred north and south exposures for uniform light distribution, are crucial [5,8,21,37,41]. Schoolchildren who required additional light were recommended to sit close to natural light sources, positioning lamps strategically behind or to the side of their stronger eye for optimal illumination. Meanwhile, schoolchildren with peripheral vision field loss may benefit from eliminating or repositioning light sources to address glare sensitivity [31]. Glare-free illumination can ideally be achieved through classroom lighting supplemented by task lighting such as desk and floor lamps and spotlighting to enhance visual acuity [29,31].

#### Color and contrast.

**Fig 3:**
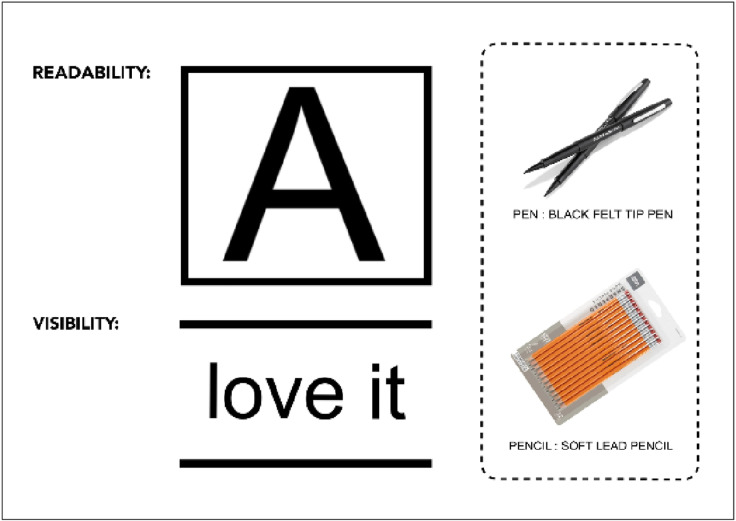
Classroom contrast guidelines. Demonstration of 100% luminance contrast principles for classroom materials and surfaces using black text on a white background.

High contrast is essential for schoolchildren with visual impairment to enhance the visibility and readability of materials. Utilizing black felt-tip pens and soft lead pencils for writing, along with employing dark print on bright white paper for handouts and highlighting lines with a black marker, underscores the importance of maximizing readability and visibility through heightened contrast [31,48], which exhibited 100% luminance contrast [33] as illustrated in (Fig 3).

**Fig 4:**
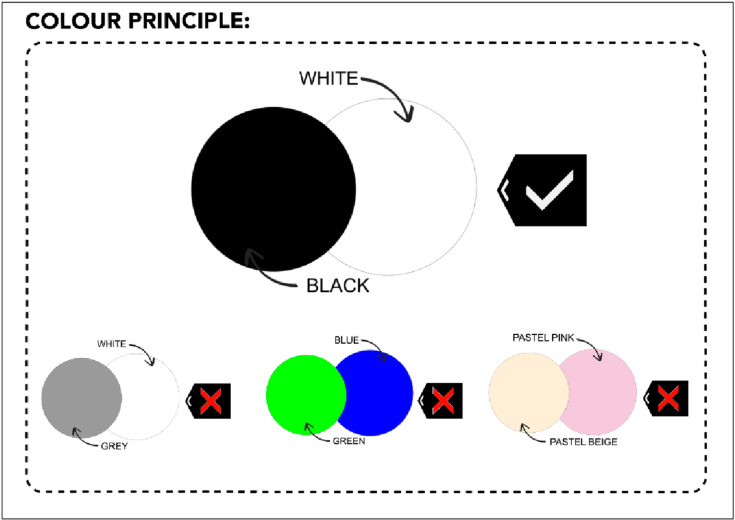
Classroom color scheme recommendations. A visual guide showing optimal color combinations and contrast levels for classroom walls, floors, and furnishings.

Additionally, it is advisable to avoid dark colour combinations such as blue, green, and pastel combinations and refrain from pairing white and grey hues with other light colours, which produce 50% luminance contrast [33]. To allow light and reduce glare, consideration of bright colour over contrasting surfaces is suggested especially for tasks in the classroom [23,24]. The recommendation of colour and contrast principles for visually impaired schoolchildren is presented in (Fig 4).

Within the classroom environment, dry-erase boards, particularly in black, are better than chalkboards due to their superior contrast, highlighting the significance of creating accessible learning environments. A clean whiteboard ensures a clear contrast between the background and the writing [24]. Writing should be considered standard print sized and typeface; Arial 14 as well as ink density [22,49]. Meanwhile, to facilitate object detection and location, floors should have a plain, non-glossy surface and a mid-toned color to distinguish them from walls. Similarly, soft furnishings should contrast with both walls and floors, preferably without intricate patterns that might hinder the identification of objects within the space [22,24,33]. Additionally, it is imperative to address surface changes such as wood or tile and consider highlighting edges for improved contrast. Temporary solutions like Duct Tape applications or permanent fixes such as painting edges can enhance visibility and safety for schoolchildren with visual impairment [22].

## Limitations and research gaps

The findings of this study might be affected by several limitations. Firstly, there could be studies that were overlooked due to bias in database selection. This scoping review focused exclusively on special educational settings and mainstream education, meaning studies examining school-based interventions implemented in other educational contexts were excluded. Furthermore, other language studies may have been unintentionally omitted, particularly since the majority of reported studies were conducted in Malaysia and were tailored to Malaysian practices.

There is a notable gap in publication regarding specific furniture designs and proper setting arrangements to meet the visual needs and demands of visually impaired schoolchildren. This indicates a need for further research and development in this area.

## Conclusion

In this scoping review, numerous key recommendations and modifications have been emerged. These recommendations aim to optimize classroom configuration, enhance visual comfort, and foster an optimal learning environment for visually impaired schoolchildren. Factors such as furniture arrangement, seating position, classroom capacity, lighting conditions, contrast, glare, and shadow management, have been identified as critical considerations. Recommendations include flexible and ergonomic furniture designs to prevent visual fatigue, regular inspections to ensure furniture suitability and safety, and tailored classroom layouts that prioritize easy navigation and literacy promotion. Moreover, proper interior lighting with uniform distribution is emphasized to enhance security, orientation, and mobility for visually impaired schoolchildren. Strategic placement of light sources, varying brightness levels and colors, and the incorporation of energy-efficient lighting options incorporated with shielding techniques are recommended to mitigate glare and flicker discomfort.

## Implications

This review examines empirical studies regarding classroom recommendations and modifications for visually impaired schoolchildren. To date, no existing review explores this research focus, addressing a crucial gap in inclusive education. By synthesizing empirical studies, this review offers insights into evidence-based practices for enhancing the educational experience of visually impaired schoolchildren. It provides educators, policymakers, and stakeholders with actionable recommendations and modifications to create a more inclusive learning environment, ultimately fostering tremendous academic success and overall well-being among this student population.

## Supporting Information

S1 File PRISMA (Preferred Reporting Items for Systematic Reviews and Meta-Analyses extension for Scoping Reviews (PRISMA-ScR), 2019 Checklist. (PDF)

S2 File A Complete Search Terms and Strategies. (PDF)

S3 File A comprehensive table of all identified studies. (XLSX)
