# Supplementary material for: Classroom settings for visually impaired schoolchildren: A scoping review
Source: PLoS One. 2025 Feb 20;20(2):e0318871. doi: 10.1371/journal.pone.0318871 (PMC11841866; doi:10.1371/journal.pone.0318871)
Supplement: S2 File — (PDF) [file pone.0318871.s002.pdf]

The search terms used for the review were:

#1. "school child*" OR pupil* OR student* OR learner*

#2. "visually impaired" OR "visual dysfunction" OR blind OR "visually disabled" OR "impaired vision" OR "vision disorder" OR "vision disability" OR "vision deterioration" OR "vision loss" OR "visual defect" OR "abnormal vision" OR "low vision" OR "reduced vision" OR "partial sight" OR "near blind" OR "subnormal vision" OR "diminished vision" OR "ocular dysfunction" OR "ocular disorder"

#3. classroom OR "learning environment"

#4. size OR dimension OR measure OR layout OR design OR organization OR setting OR light* OR illumination OR contrast OR "seating position"

#5. 1 AND 2 AND 3 AND 4

Each search term was associated with various MeSH terms. For "school child," terms such as 'pupil,' 'learner,' or 'student' were included. The search terms for 'visually impaired' encompassed 'low vision,' 'visual dysfunction,' or 'blind.' MeSH terms relevant to 'classroom size' included 'dimension' or 'measure,' and for 'classroom light' or 'lighting,' 'classroom illumination' was considered. However, both 'classroom contrast' and 'classroom seating' were not connected to other MeSH terms.
